# Dogs Licks Are Not Benign: Pasturella Multocida Bacteremia From Household Dog

**DOI:** 10.7759/cureus.58554

**Published:** 2024-04-18

**Authors:** Katherine Hageboeck Shepherd, Mark Colantonio, Matthew Santer, Haseeb Mahmud, Brooke Shannon

**Affiliations:** 1 Internal Medicine, West Virginia University School of Medicine, Morgantown, USA

**Keywords:** pressor support, septic shock, cellulitis, gram negative bacteremia, pasteurella multocida

## Abstract

*Pasturella (P.) multocida* is a gram-negative coccobacilli commonly colonized in the oral, nasopharyngeal, and upper respiratory tracts of animals. Infections due to *P. multocida *range in severity, and symptoms largely depend on underlying immune status and co-morbid conditions. Widely known, the transmission of *P. multocida *is commonly thought to occur through biting and skin breakage alone. However, multiple studies have highlighted instances of severe complications secondary to transmission through the passage of *P. multocida *through animal licking alone without skin disruption. Here, we present a case of a nonagenarian female presenting with septic shock secondary to *P. multocida *with the source of transmission found to be secondary to the patient's dog licking her chronic leg wounds. We also highlight other instances of similar transmission through a literature review, including common treatment courses. We aim to raise awareness of common transmissions of bacteria, specifically *P. multocida*, along with broadening differentials when one presents with skin and soft tissue infections.

## Introduction

*Pasturella (P.) multocida* is a gram-negative coccobacilli bacteria commonly found with household pets [1]. *P. multocida* has five associated serotypes, with serotypes A and D being the most likely to cause infection [1]. Although most associated with dogs, cats have the highest transmission rate of *P. multocida*,* *at 70-90%, with dogs coming in second with a rate of 20-50% [1]. Studies have also linked the transmission of *Pasteurella* with other organisms, including rats, horses, and even rabbits [1]. Transmission is commonly from direct contact with animals, including bites, scratches, or contact with oral, nasopharyngeal, or upper respiratory tract secretions [1]. However, transmission has also been documented from licking alone [1]. Surprisingly, one study revealed bloodstream infections were most commonly observed without overt animal bites, but rather through direct skin contact with animal secretions, whereas localized soft tissue infections were more commonly observed with traumatic biting and skin breakage [2]. In addition, this same study found patients without an animal bite were more likely to have a longer length of stay, as well as require an intensive care unit (ICU) level of care [2]. Due to the high rate of transmission, even without breaking the skin barrier, clinicians should have a high suspicion for *P. multocida*, especially in patients with household pets. 

A common complication of *P. multocida* inoculation includes erythema and, in some cases, drainage, leading to non-purulent or purulent cellulitis [1]. This complication typically develops within 24 hours of transmission [1]. In more severe cases, complications include peritonitis, tubo-ovarian abscess formation, and necrotizing fasciitis [1,3]. Underlying patient comorbidities can influence the disease course of *P. multocida*, making specific patient populations more susceptible to infection. Chatelier et al. identified cirrhosis, malignancy, and immunosuppressive therapy as more common morbidities associated with poor outcomes [4]. Due to the commonality of household pets and the propensity to form serious complications, early identification and treatment are necessary. Here, we present a case of a nonagenarian female presenting with endorsements of weakness or fatigue, later found to be associated with a concurrent *P. multocida* infection.

## Case presentation

**Figure 1:**
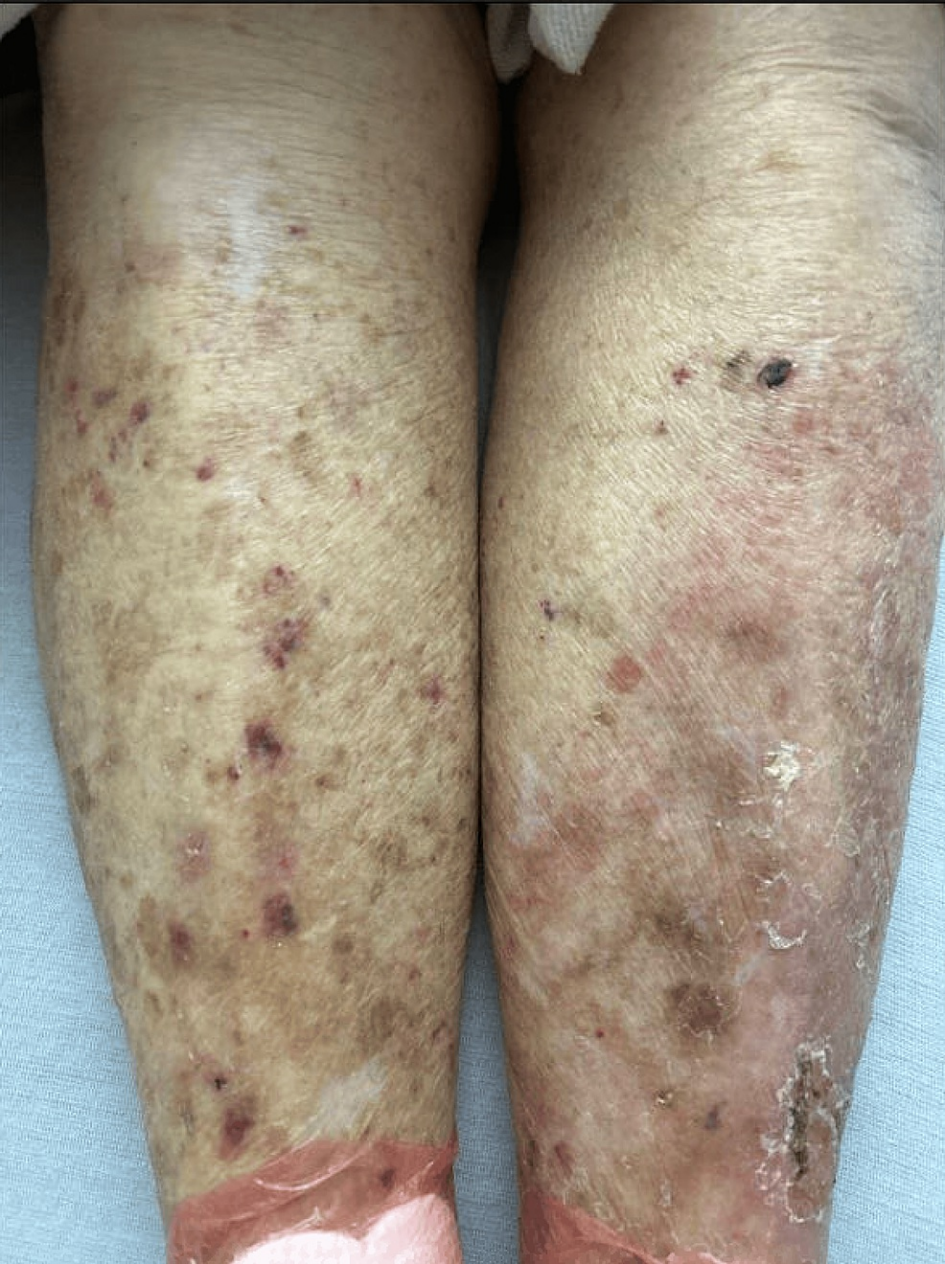
Bilateral lower extremities at presentation to the emergency department Visible erythema without abscess or drainage can be visualized on the left lower extremity. Chronic scarring and healing wounds can also be visualized on the extremity.

**Figure 2:**
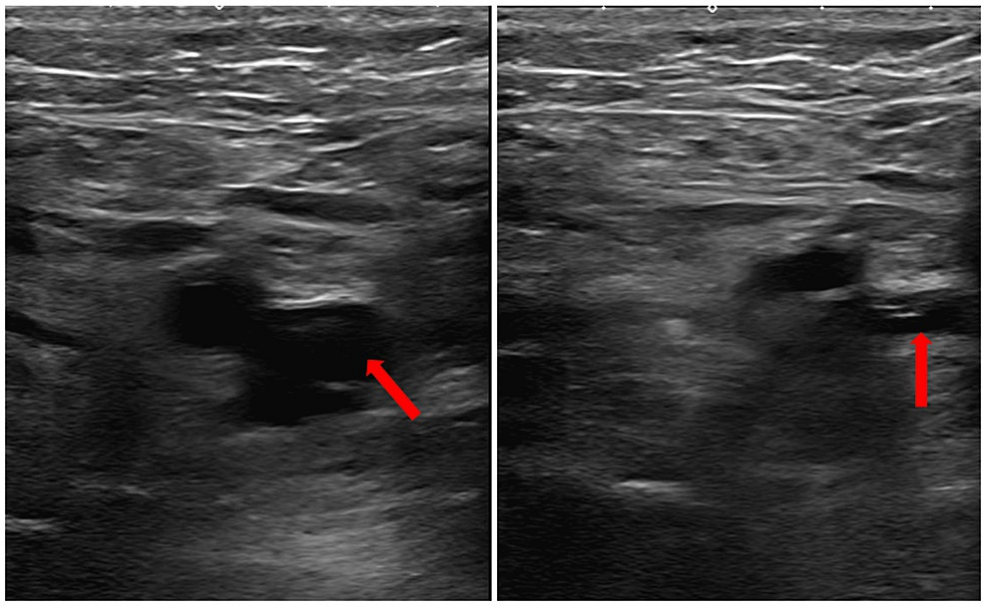
Duplex ultrasonography of the left lower extremity Venous system denoted by a red arrow with subsequent evidence of collapsibility

The patient was a nonagenarian female with a past medical history of hypertension who presented to the emergency department (ED) with endorsements of weakness for several days and an unwitnessed fall. On initial presentation, the patient was hypotensive, tachycardic, tachypneic, and afebrile. Physical exam was remarkable for confusion, tachycardia, and chronic skin breakdown on bilateral lower extremities with left lower extremity erythema (Figure 1). Initial blood cultures were obtained. Duplex ultrasonography was also obtained of bilateral lower extremities to rule out deep vein thrombosis in the setting of erythema, warmth, and increasing pain (Figure 2). The patient was ultimately given 2 L of normal saline and started on vancomycin, cefepime, and flagyl for empiric antimicrobial coverage. Due to persistent hypotension, a central line was placed, and the patient was started on Levophed. The patient was transferred to the ICU due to hemodynamic instability requiring pressure support.

Initial laboratory work revealed a white blood count (WBC) of 9.0 x 10^3/uL, lactic acid of 3.8 mmol/L, unremarkable urinalysis, and negative respiratory viral panel. C-reactive protein (CRP) was elevated at 50.5 mg/L. Troponin was elevated to 1068 ng/L at admission with a peak of 4172 ng/L. The electrocardiogram (EKG) was unremarkable for acute ischemic changes. Chest X-ray revealed a hazy opacity throughout the right lung.

The patient remained on pressure support with Levophed for approximately 12 hours and was ultimately weaned off as pressures subsequently improved. The central line catheter was removed. Previously collected blood cultures collected from two sites, including an aerobic and anaerobic bottle, grew gram-negative coccobacilli from both sites. Further speciation revealed *Pasteurella multocida*. Upon further discussion with the patient, the patient stated her dog, a shih tzu, licks her chronic leg wounds daily. Due to the blood culture growth of *P. multocida*, antibiotics were transitioned to Unasyn. Cardiology was consulted due to significantly elevated troponin at admission in the setting of hypotension. A transthoracic echocardiogram (TTE) was recommended and revealed moderate to severe mitral regurgitation, a new, mildly depressed left ventricular ejection fraction of 45%, and severe pulmonary hypertension. After a risk-benefit discussion with the patient and family, the decision was made to perform a transesophageal echocardiogram (TEE) for better visualization of the mitral valve to rule out valvular vegetation. TEE demonstrated a recovered left ventricular ejection fraction of 55% with an absence of vegetation on the mitral valve. The acute drop and recovery in ejection fraction in the setting of elevated troponin was likely secondary to stress-induced cardiomyopathy in the setting of demand ischemia. The patient was continued on antibiotics for 14 days after the first set of blood cultures were negative for further bacterial growth. Unasyn was transitioned to Augmentin after day 10 of intravenous antibiotics. After seven days of appropriate antibiotic therapy, CRP decreased to 13.2 mg/L and WBC decreased to 4.2x10^3/uL. The patient ultimately completed antibiotic therapy and recovered without further complications.

## Discussion

*P. multocida* is a gram-negative bacteria commonly responsible for skin and soft tissue infections secondary to dog and/or cat exposure [1]. Commonly thought to be transmitted via biting, our case highlights an uncommon route of transmission through non-traumatic contact with a household pet. Skin and soft tissue infections, especially in patients with chronic open-skin wounds, such as our case, should heighten the suspicion of *P. multocida* infection. Philip et al. described a case of an elderly female who ultimately developed a prosthetic joint infection due to *P. multocida* [5]. Transmission in this case was thought to be secondary to multiple pet dogs licking the patient's chronic, non-healing hip wound after replacement [5]. The patient ultimately underwent multiple revisional arthroplasties to resolve the infection [5]. Special caution about pet exposure should be taken in vulnerable populations, especially the very young and elderly. Ryan et al described a more severe case of *P. multocida* meningitis in a 12-day-old baby due to oral contact with oral and nasopharyngeal secretions from a household pet [6]. In the elderly and immunocompromised, respiratory tract infections are more common secondary to *P. multocida* [7]. Myers et al. described three cases of severe respiratory tract infection leading to *P. multocida* pneumonia. In each case, transmission of *P. multocida* was due to pet exposure in the setting of palliative pet care for a chronic illness [7]. This case highlights the importance of physicians taking part in a risk-benefit discussion with palliative patients, even in cases as benign as palliative pet care [7]. Taking a deeper dive to determine susceptible patient populations, Chatelier et al. performed a retrospective chart review of *P. multocida* cases from 2008 to 2017 [4]. They determined the populations most susceptible to complications included those with underlying cirrhosis, malignancy, and immunosuppression [4]. Males were also more likely to develop complications compared to females [4]. Our case, along with the others outlined, highlights the importance of caution when household pets are exposed to populations vulnerable to infection, including infants, the elderly, the immunocompromised, and especially those with chronic, open wounds.

Once identified, *P. multocida* is typically susceptible to a variety of antibiotic classes [8]. To date, there is no clear evidence recommending one antibiotic class over another for monotherapy treatment of *P. multocida* [9]. Treatment choice is dependent on clinical presentation, clinical course, and the type of infection being treated. For patients presenting with skin and soft tissue infections secondary to an animal bite or exposure, such as in our case, empiric polymicrobial coverage is recommended [9]. In our case, empiric vancomycin, cefepime, and flagyl were initially started for broad-spectrum polymicrobial coverage. Antibiotic therapy was then narrowed to IV Unasyn once blood cultures resulted in *P. multocida*. Management was consistent with prior case reports and recommendations [1]. In more severe cases, specifically those diagnosed with septic arthritis and/or osteomyelitis secondary to *P. multocida*, prolonged therapy is recommended for four to six weeks [10]. Narsana et al. described a case of septic shock secondary to *P. multocida* infection that was ultimately treated with a 25-day course of imipenem-cisplatin [11]. Although commonly treated with penicillin, this case highlights the importance of escalation and antibiotic care in the presence of clinical worsening [11]. Penicillin resistance is rare; however, site-specific resistance, specifically to the wrist, has been noted and is associated with worse outcomes [8,12].

## Conclusions

A broad differential is important when treating skin and soft tissue infections. *P. multocida *should be suspected in the setting of close contact with household pets, including cats and dogs, even without traumatic bite wounds. Our case highlights the transmission of *P. multocida* through contact with oral pet secretions via licking. We also highlight a successful treatment plan for *P. multocida* with the initiation of broad-spectrum antibiotics followed by a transition to Unasyn and subsequent oral Augmentin. Future studies should aim to raise awareness of other routes of bacterial transmission that may not be suspected, especially in susceptible patient populations.
